# Draft Genome Sequence of *Enterococcus* sp. Strain HSIEG1, Isolated from the Human Small Intestine

**DOI:** 10.1128/genomeA.01013-13

**Published:** 2013-12-12

**Authors:** Bartholomeus van den Bogert, Jos Boekhorst, Eddy J. Smid, Erwin G. Zoetendal, Michiel Kleerebezem

**Affiliations:** Top Institute Food and Nutrition (TIFN), Wageningen, the Netherlandsa; Laboratory of Microbiology, Wageningen University, Wageningen, the Netherlandsb; Centre for Molecular and Biomolecular Informatics, Radboud University Medical Centre, Nijmegen, the Netherlandsc; NIZO Food Research BV, Ede, the Netherlandsd; Laboratory of Food Microbiology, Wageningen University, Wageningen, the Netherlandse; Host-Microbe Interactomics Group, Wageningen University, Wageningen, the Netherlandsf

## Abstract

*Enterococcus* sp. strain HSIEG1 was isolated from the human small intestine. Its draft genome predicts a broad carbohydrate fermentation capability, which matches well with the observed physiological characteristics of this strain. This metabolic flexibility is expected to be of importance for survival and growth in the small intestinal habitat.

## GENOME ANNOUNCEMENT

The human small intestine is commonly predominated by facultative anaerobes, such as *Streptococcus* spp. (1–5). The relative abundances of other lactic acid bacteria, including enterococci, are generally low (4, 6) (M. M. Leimena, B. van den Bogert, J. Boekhorst, E. J. Smid, E. G. Zoetendal, and M. Kleerebezem, unpublished data) but can in some cases constitute a sizeable fraction of the overall microbial community in this ecosystem (7, 8). In an effort to obtain representative bacterial isolates from the small intestinal ecosystem, seven *Enterococcus* lineages were recovered from ileostoma effluent samples. These isolates belonged to the *Enterococcus avium*, *Enterococcus faecium*, *Enterococcus faecalis*, and *Enterococcus gallinarum* groups, which demonstrates the substantial level of phylogenetic richness of enterococci in the small intestine (9).

The draft genome sequence of a representative isolate from the lineage belonging to the *E. gallinarum* species group, *Enterococcus* sp. strain HSIEG1, was obtained by sequencing of 3-kb mate-pair libraries using 454 GS FLX (Roche) technology in combination with titanium chemistry and Illumina HiSeq 2000 technology (GATC Biotech, Konstanz, Germany). A total of 153,444 pyrosequencing reads were assembled using the Celera Assembler version 6.1 (http://sourceforge.net/apps/mediawiki/wgs-assembler/index.php?title=Main_Page) into 158 contigs, which were placed in their likely order by employing the 10,557,832 paired reads from Illumina sequencing using the SSPACE software version 1.1 (10). This pseudoassembly was manually screened for inconsistencies using the Artemis Comparison Tool (11). The genome was annotated using the RAST server (12). The final assembly of the *Enterococcus* sp. HSIEG1 genome contains 3,447,751 bp, with an average ~300-fold coverage, a G+C content of 40.45%, and 3,901 predicted protein-coding genes.

Almost 10% of the coding capacity encountered in the genome of HSIEG1 is dedicated to genes assigned to functions related to carbohydrate transport and metabolism. The HSIEG1 genome encodes single copies of the generic cytoplasmic factors enzyme I (EI) and phospho-carrier protein (HPr), which are involved in phosphotransfer of >30 phosphotransferase system (PTS) transporter functions with predicted specificities that include glucose/maltose, mannose, fructose, galactose, lactose, sucrose, cellobiose, and β-glucosides. Moreover, the genome encodes several ABC sugar transporters, including those predicted to be involved in maltose/maltodextrin transport. In addition to these transport-associated functions, the genome also encodes the necessary pathways to metabolize these sugars as well as arabinose, ribose, and xylose. HSIEG1 has the capacity to ferment all these sugars, showing that the genome predictions are in good agreement with the observed physiological characteristics. The metabolic flexibility of HSIEG1 may be of relevance for its survival in a nutrient-fluctuating environment, such as in the small intestine (13).

Following the transport and primary conversions of carbohydrates, the genome is predicted to encode the canonical enzymes of the glycolytic conversion pathway, which is the main energy-generating pathway in this species. The pyruvate dissipation pathways predicted for HSIEG1 include the capacity to produce l-lactate and several other fermentation metabolites, like formate, acetate, ethanol, acetoin, and 2,3-butanediol.

### Nucleotide sequence accession numbers.

This whole-genome shotgun project has been deposited at DDBJ/EMBL/GenBank under the accession no. ASKG00000000. The version described in this paper is version ASKG01000000.
